# Utility of the combination of DAT SPECT and MIBG myocardial scintigraphy in differentiating dementia with Lewy bodies from Alzheimer’s disease

**DOI:** 10.1007/s00259-015-3146-y

**Published:** 2015-08-02

**Authors:** Soichiro Shimizu, Kentaro Hirao, Hidekazu Kanetaka, Nayuta Namioka, Hirokuni Hatanaka, Daisuke Hirose, Raita Fukasawa, Takahiko Umahara, Hirohumi Sakurai, Haruo Hanyu

**Affiliations:** Department of Geriatric Medicine, Tokyo Medical University, 6-7-1 Nishishinjuku, Shinjuku-ku, Tokyo 160-0023 Japan

**Keywords:** ^123^I-FP-CIT, DAT, SPECT, MIBG myocardial scintigraphy, Alzheimer’s disease, Dementia with Lewy bodies

## Abstract

**Purpose:**

^123^I-2β-Carbomethoxy-3β-(4-iodophenyl)-*N*-(3-fluoropropyl) nortropane (^123^I-FP-CIT) dopamine transporter single photon emission computed tomography (DAT SPECT) and ^123^I-metaiodobenzylguanidine (MIBG) myocardial scintigraphy can be used to assist in the diagnosis of patients with dementia with Lewy bodies (DLB). We compared the diagnostic value of these two methods in differentiating DLB from Alzheimer’s disease (AD). Furthermore, we evaluated whether a combination of DAT SPECT and MIBG myocardial scintigraphy would provide a more useful means of differentiating between DLB and AD.

**Methods:**

Patients with AD (*n* = 57) and patients with DLB (*n* = 76) who underwent both DAT SPECT and MIBG myocardial scintigraphy were enrolled. The sensitivity, specificity, and accuracy of both methods as well as their combination for differentiating DLB from AD were calculated. Moreover, we examined whether symptoms of the patients with DLB were associated with the patterns of the abnormalities displayed on DAT SPECT and MIBG myocardial scintigraphy.

**Results:**

The sensitivity and specificity of differentiating DLB from AD were 72.4 and 94.4 % by the heart to mediastinum ratio of MIBG uptake, 88.2 and 88.9 % by the specific binding ratio on DAT SPECT, and 96.1 and 90.7 % by their combination, respectively. The combined use of DAT SPECT and MIBG myocardial scintigraphy enabled more accurate differentiation between DLB and AD compared with either DAT SPECT or MIBG myocardial scintigraphy alone. There was a significantly higher frequency of parkinsonism in the abnormal DAT SPECT group than the normal DAT SPECT group. On the other hand, there was a higher frequency of the appearance of rapid eye movement (REM) sleep behavior disorder in the abnormal MIBG uptake group than the normal MIBG uptake group.

**Conclusion:**

These results suggested that using a combination of these scintigraphic methods is a useful and practical approach to differentiate DLB from AD.

## Introduction

Dementia with Lewy bodies (DLB) is recognized as the second most common cause of degenerative dementia in older people, following Alzheimer’s disease (AD). In some cases, the clinical differentiation of patients with DLB from those with AD may be difficult because of overlapping clinical and pathological features. The importance of accurate identification of patients with DLB lies particularly in its pharmacological management, with favorable responsiveness to cholinesterase inhibitors but severe sensitivity to the adverse effects of neuroleptic agents [1].

The first consensus clinical diagnostic criteria for DLB that were developed [2] had high specificity for the diagnosis of probable DLB, but poor sensitivity [3]. In view of these difficulties the consensus criteria were revised [4], with the addition of new features to improve the diagnosis of DLB. Abnormal findings on dopamine transporter (DAT) imaging were considered the most important among the various neuroimaging features listed as one of the suggestive features of DLB, whereas findings from other neuroimaging techniques, including ^123^I-metaiodobenzylguanidine (MIBG) myocardial scintigraphy were listed only as supportive of DLB (commonly present in DLB but not proven to have diagnostic specificity).

^123^I-2β-Carbomethoxy-3β-(4-iodophenyl)-*N*-(3-fluoropropyl) nortropane (^123^I-FP-CIT), a ligand that binds to the presynaptic DAT, can be used to analyze the integrity of the nigrostriatal projection pathway. ^123^I-FP-CIT DAT single photon emission computed tomography (SPECT) has been used in a large number of trials to identify the in vivo loss of DATs in the striatum of patients with presynaptic parkinsonism [5, 6]. Previous studies showed that DAT SPECT substantially enhanced the accuracy of the diagnosis of DLB compared with clinical criteria alone and has a high diagnostic accuracy in differentiating DLB patients from non-DLB patients [7–16].

On the other hand, recent studies have indicated that MIBG myocardial scintigraphy is able to detect early disturbances of the sympathetic nervous system in DLB, independently of the duration of disease and autonomic failure, and provides diagnostic information useful for differentiating DLB from AD [17–24].

The usefulness of both DAT SPECT and MIBG myocardial scintigraphy for the diagnosis of DLB has recently been suggested by two meta-analysis studies [25, 26]. Moreover, the utility of the combination of DAT SPECT and MIBG myocardial scintigraphy was reported in patients with DLB and Parkinson syndrome (PS) [14, 15, 27–29]. Furthermore, the use of ^123^I-FP-CIT was approved by the Japanese Ministry of Health, Labour and Welfare in February 2014.

In the present study, we performed both DAT SPECT and MIBG myocardial scintigraphy in patients with DLB and AD, and compared the diagnostic value of these two methods in differentiating DLB from AD. We also examined whether a combination of DAT SPECT and MIBG myocardial scintigraphy would provide a more useful means of differentiating between DLB and AD compared with either of the two methods alone. Moreover, we examined whether the particular symptoms of the DLB patients would be associated with the abnormalities observed on DAT SPECT and MIBG myocardial scintigraphy. To our knowledge, this is the first study to evaluate the diagnostic value of a combination of these two methods in differentiating DLB from AD.

## Materials and methods

### Patients

A total of 133 outpatients with AD or DLB from the Memory Disorder Clinic at the Department of Geriatric Medicine, Tokyo Medical University, were enrolled in this study from March 2014 until September 2014. They had a dementia severity of 1 (mild) or 2 (moderate) based on the Clinical Dementia Rating [30] and Mini-Mental State Examination (MMSE) [31] scores between 14 and 26. All subjects underwent both DAT SPECT and MIBG myocardial scintigraphy. The interval between undergoing the two methods of imaging was less than 2 months. Of the 133 patients, 57 had a diagnosis of probable AD based on the National Institute of Neurological and Communicative Disorders and Stroke and Alzheimer’s Disease and Related Disorders Association (NINCDS-ADRDA) criteria [32] and the other 76 had a diagnosis of probable (36 patients) and possible DLB (40 patients) based on the consortium on DLB international workshop criteria [4], except for low DAT uptake in the basal ganglia, which was also included because one of the aims of this study was to determine whether DAT SPECT is useful for the diagnosis of DLB.

All patients underwent general physical, neurological, and psychiatric examinations, extensive laboratory tests, and computed tomography (CT) or magnetic resonance imaging (MRI) to establish a clinical diagnosis and to exclude other potential causes of dementia. None of the subjects had any history of cerebrovascular disease, other degenerative diseases, infarction in the region of the basal ganglia or intracranial lesions on brain MRI, thyroid disease, diabetes mellitus, or previous relevant cardiac disease, nor were taking any medications known to interact with the striatal binding of ^123^I-FP-CIT (e.g., cocaine, amphetamines, bupropion, selective serotonin reuptake inhibitors, etc.) [33, 34] or that affect MIBG accumulation [35].

The Hachinski ischemic score [36] was less than 4 in all patients. Based on the UK Parkinson’s Disease Society Brain Bank criteria [37], parkinsonism was defined as the presence of bradykinesia, associated with one or more of the following three features: tremor, rigidity, or postural instability.

None of the patients with probable AD had fluctuating cognition, visual hallucinations, parkinsonism, or rapid eye movement (REM) sleep behavior disorder (RBD) as determined by three geriatric neurologists (H.H., T.U., and S.S.).

This study was approved by the Ethics Committee of Tokyo Medical University. Informed consent was obtained from all subjects (either the patients themselves or their closest relative) before entry, following a detailed explanation of the study’s aim. In accordance with the research plan, the fees of MIBG myocardial scintigraphy and DAT SPECT examination for the patients with AD were paid for using the research funds of our department. All procedures were in accordance with the ethical standards on human investigation and with the principles of the Declaration of Helsinki.

### Image analysis

#### ^123^I-MIBG myocardial scintigraphy

After the patient had rested for 15 min in the supine position, 111 MBq of ^123^I-MIBG was injected intravenously. Early and delayed SPECT were performed at 20 min and 4 h after the injection, respectively. Planar imaging for 5 min in the anterior projection was performed during SPECT automatically. Planar scan and SPECT were performed with a dual-head gamma camera equipped with a low-energy, high-resolution parallel-hole collimator (PRISM 2000VP, Picker). After the scatter correction, relative organ uptake was determined by setting the region of interest (ROI) on the anterior view [38]. The heart to mediastinum (H/M) ratio was calculated by dividing the count density of the left ventricular ROI by that of the mediastinal ROI, according to the standard method described previously [17, 39]. The normal H/M ratios in the early phase and the delayed phase as well as the washout ratios obtained from eight normal elderly controls in our institute (three men and five women, mean age 76.5 ± 5.8 years) were 2.56 ± 0.37, 2.53 ± 0.38, and 32.90 ± 10.26, respectively. Values were considered abnormal if they were less than 2 SDs below the control mean. For the comparison study, H/M ratios calculated from the ROI counts obtained by delayed SPECT were used for analysis, because delayed scans display the neuronal uptake of MIBG more explicitly [17].

#### DAT SPECT imaging and specific binding ratio analysis

Three hours after injection of approximately 185 MBq of ^123^I-FP-CIT, projection data were obtained in a 128 × 128 matrix on a Siemens Symbia T16 mounted with low- to medium-energy general purpose (LMEGP) collimators. Projection data were acquired for 28 min. Data were reconstructed by ordered subset expectation maximization (OSEM) method (iteration 8, subset 6) using Flash 3D software (Siemens) and corrected for attenuation by CT. The specific binding ratio (SBR) was semiquantitatively calculated using DAT VIEW software (Nihon Medi-Physics, Tokyo, Japan) based on Bolt’s method, as described in detail elsewhere [40]. For this study, we used SBR as the mean value of the right and left SBRs. The control group for DAT SPECT consisted of 18 subjects without any present or previous neurological disease (7 men and 11 women, mean age 78.6 ± 6.5 years). The mean SBR of the controls was 5.84 ± 0.83. Values were considered abnormal if they were less than 2 SDs below the control mean.

### Statistical analysis

Values were expressed as means ± SD and analyzed by Student’s *t* test, χ^2^ test, and one-way analysis of variance. A *p* value of less than 0.05 was considered to indicate a statistically significant difference between the two groups. The sensitivity and specificity of the respective diagnostic index (H/M ratios of MIBG uptake in the delayed phase, SBR on DAT SPECT, and combined DAT SPECT and MIBG myocardial scintigraphy) for the differentiation between DLB and AD were assessed using receiver-operating characteristic (ROC) analysis. For the combined use of DAT SPECT and MIBG myocardial scintigraphy, we developed the combined DAT*MIBG index, defined as (SBR*H/M in the delayed phase). The cutoff values (mean − 2 SD) were 1.82, 1.77, 12.38, and 4.18 for the H/M in the early phase, the H/M in the delayed phase, washout ratio, and SBR, respectively. We adopted the best value in ROC analysis as the cutoff value (10.18) of the DAT*MIBG index. All data were statistically analyzed using MedCalc software (version 13.3.0.0, MedCalc Software, Mariakerke, Belgium).

## Results

Table 1 shows the characteristics of the patients. No significant differences in the two groups were found in terms of age, length of education, duration of disease, and MMSE scores. The number of women was significantly higher in the AD group (*p* < 0.01).

**Table 1:** Characteristics of the patients

	AD (*n* = 57)	DLB (*n* = 76)
Age (years)	81.4 ± 6.1	80.2 ± 4.9
Sex (male/female)	10/47*	42/34
Length of education (years)	10.3 ± 3.2	11.8 ± 2.8
Duration of disease (years)	2.8 ± 1.2	3.2 ± 0.9
MMSE score	22.5 ± 5.4	22.4 ± 4.8

*MMSE* Mini-Mental State Examination

*p < 0.01

**Fig. 1:**
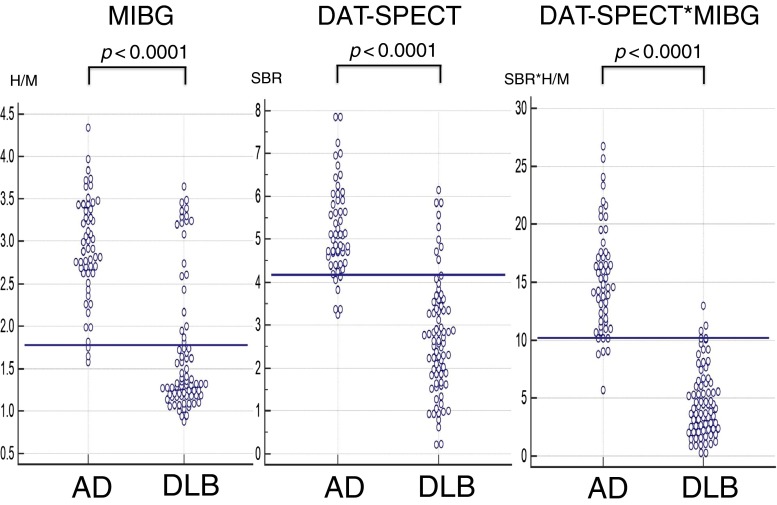
Scatter plots of the H/M ratio on MIBG myocardial scintigraphy in the delayed phase (*left*), SBR on DAT SPECT (*middle*), and DAT*MIBG index (*right*) in patients with DLB and AD. Cutoff lines were set at 1.77 (2 SDs below the control, *right*), 4.18 (2 SDs below the control, *middle*) and 10.18 (the best value in ROC analysis, *left*)

Mean H/M ratios of MIBG uptake in the early phase (3.2 ± 0.5 vs 2.2 ± 0.8, *p* < 0.0001, cutoff 1.82) and delayed phase (2.9 ± 0.6 vs 1.7 ± 0.8, cutoff 1.77, *p* < 0.0001, Fig. 1, left) were significantly lower and mean washout ratios (21.7 ± 10.0 vs 38.3 ± 10.2, cutoff 12.38, *p* < 0.0001) were significantly higher in patients with DLB than in patients with AD. ROC analysis demonstrated that there was no significant difference in diagnostic accuracy among these three ratios. The area under the curve was 0.864 for the delayed phase, 0.859 for the washout ratio, and 0.835 for the early phase.

Mean SBRs on DAT SPECT were markedly lower in patients with DLB than in those with AD (5.2 ± 1.0 vs 2.7 ± 1.3, cutoff 4.18, *p* < 0.0001, Fig. 1, middle). Mean values of the DAT*MIBG index (described in the “Materials and methods” section) were significantly lower in patients with DLB than in those with AD (15.3 ± 4.5 vs 4.6 ± 3.0, cutoff 10.18, *p* < 0.0001, Fig. 1, right). The sensitivity and specificity in differentiating DLB from AD were 72.4 and 94.4 % by the H/M ratio of MIBG uptake in the delayed phase, 88.2 and 88.9 % by the SBR on DAT SPECT, and 96.1 and 90.7 % by the DAT*MIBG index, respectively.

**Fig. 2:**
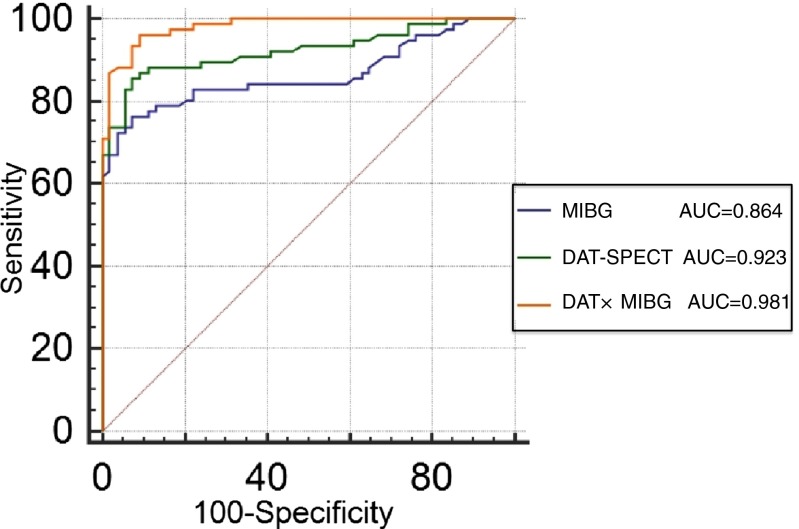
ROC curves to differentiate DLB from AD. The combination of DAT SPECT and MIBG myocardial scintigraphy enabled more accurate differentiation between DLB and AD. *AUC* area under the curve

The area under the curve was 0.864 for the H/M ratio of MIBG myocardial scintigraphy in the delayed phase, 0.923 for DAT SPECT, and 0.981 for the combined use of these two methods (Fig. 2). The combined use of the two methods enabled more accurate differentiation of DLB from AD than either DAT SPECT or MIBG myocardial scintigraphy alone (DAT SPECT vs MIBG, *p* = 0.170; DAT SPECT and MIBG vs MIBG, *p* < 0.001; DAT SPECT and MIBG vs DAT SPECT, *p* = 0.012).

**Fig. 3:**
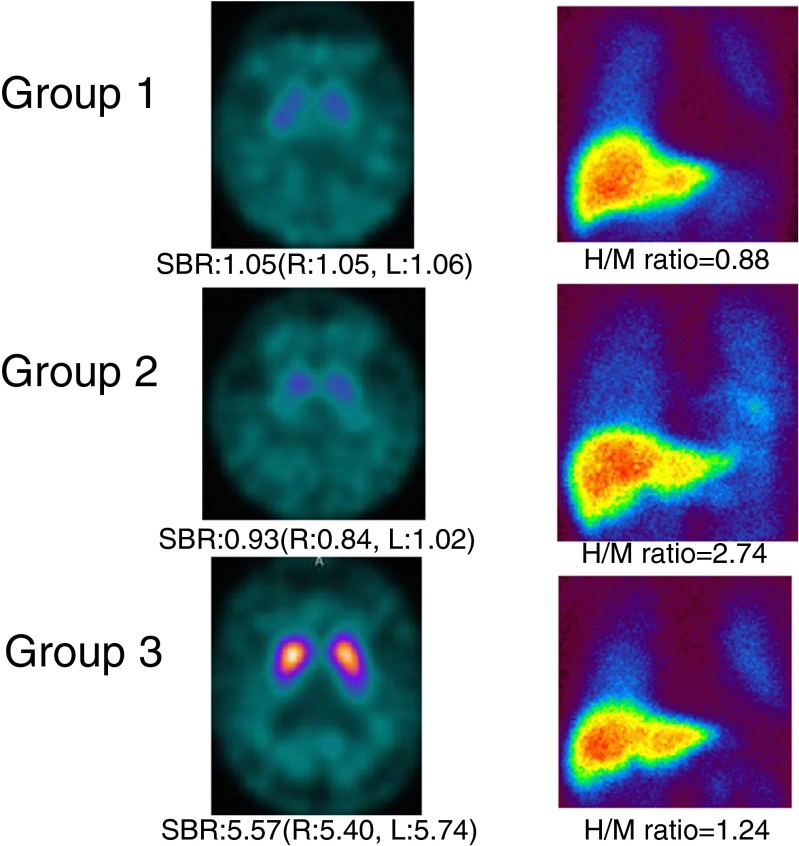
Example images of DAT SPECT and MIBG myocardial scintigraphy of patients with DLB. Group 1: low uptakes on both DAT SPECT and MIBG myocardial scintigraphy (*top*). Group 2: low uptake on DAT SPECT and normal on MIBG myocardial scintigraphy (*middle*). Group 3: normal on DAT SPECT and low uptake on MIBG myocardial scintigraphy (*bottom*)

Patients with DLB were categorized into three different groups by the combined use of DAT SPECT and MIBG myocardial scintigraphy. Figure 3 shows representative DAT SPECT and MIBG myocardial scintigraphy images of patients from the three different groups. Forty-six patients showed reduced tracer uptake on both DAT SPECT and MIBG myocardial scintigraphy (Fig. 3, top). Twenty-one patients showed decreased DAT uptake but normal MIBG uptake (Fig. 3, middle). Nine patients showed normal DAT uptake and decreased MIBG uptake (Fig. 3, bottom).

Table 2 shows a summary of the symptoms observed in patients with DLB in the three groups. The appearance of parkinsonism was significantly more frequent in patients from groups 1 and 2 that had abnormal DAT SPECT data, compared with patients from group 3 that had normal DAT SPECT data (group 1 vs group 3: *p* < 0.001; group 2 vs group 3: *p* < 0.001). On the other hand, the appearance of RBD in patients of groups 1 and 3 that had abnormal MIBG uptake was significantly more frequent compared with patients from group 2 that had normal MIBG uptake (group 1 vs group 2: *p* < 0.01; group 3 vs group 2: *p* < 0.05).

**Table 2:** DAT SPECT and MIBG myocardial scintigraphy in patients with DLB

	Group 1	Group 2	Group 3
	Low DAT uptake, low MIBG uptake	Low DAT uptake, normal MIBG uptake	Normal DAT uptake, low MIBG uptake
Total (*n*)	46	21	9
Age (mean ± SD)	80.2 ± 5.4	80.3 ± 4.2	79.6 ± 4.6
Sex (male/female)	26/20	11/10	5/4
Prob./poss.^a^	28 (61 %)/18 (39 %)	6 (29 %)/15 (71 %)	2 (22 %)/7 (78 %)
SBR on DAT SPECT	2.29 ± 1.03###	2.48 ± 0.88###	5.38 ± 0.58
H/M of MIBG	1.25 ± 0.20*** #	2.86 ± 0.61###	1.47 ± 0.21
MMSE	23.2 ± 4.3	21.0 ± 5.1	21.9 ± 6.0
Hoehn and Yahr score	2.2 ± 0.9	2.2 ± 1.2	1.0 ± 1.4
Parkinsonism, *n* (%)	38 (83 %)###	18 (86 %)###	2 (22 %)
Hallucination, *n* (%)	21 (46 %)	5 (24 %)	3 (33 %)
Fluctuation, *n* (%)	11 (24 %)	3 (14 %)	2 (22 %)
RBD, * n* (%)	23 (50 %)**	2 (10 %)#	4 (44 %)

*MMSE* Mini-Mental State Examination

*p < 0.05; **p < 0.01; ***p < 0.001 (vs low DAT uptake/normal MIBG uptake)

#*p* < 0.05; ##*p* < 0.01; ###*p* < 0.001 (vs normal DAT uptake/low MIBG uptake)

^a^Diagnosed as having DLB, as described in the manuscript

## Discussion

In agreement with previous studies [7–26], we confirmed that a reduction in both striatal DAT uptake and cardiac MIBG uptake are characteristic features of DLB, and the combined use of DAT SPECT and MIBG myocardial scintigraphy was more useful for differentiating DLB from AD, compared with either of these two methods alone. Moreover, we found the presence of parkinsonism at a significantly higher frequency in patients with DLB who had low DAT uptake. On the other hand, RBD was frequently observed in patients with DLB who showed abnormalities on MIBG myocardial scintigraphy.

Previous studies in which neuropathological autopsy was performed [10, 41] suggested that DAT imaging assists in the diagnosis of patients with DLB. A recent meta-analysis study reported a pooled sensitivity of 86.5 % and a specificity of 93.6 % for the differentiation of DLB from non-DLB using DAT SPECT [26]. In our present study, the sensitivity and specificity of differentiating DLB from AD by DAT SPECT were 88.2 and 88.9 %, respectively, which were consistent with the results of previous studies (sensitivities of 80 % and specificities of 90–94 %) [7–9].

In DLB, the loss of dopaminergic cells is accompanied by the loss of DATs (presynaptic receptors). A previous in vitro study suggested that the loss of DAT and the loss of striatal dopamine content are linearly correlated [42]. Our result showing the significantly frequent appearance of parkinsonism in patients with DLB who showed abnormalities on DAT SPECT was consistent with this previous in vitro study. In patients with PD, the severity of motor symptoms often correlates inversely with DAT density [43, 44]. However, this association may be different in patients with DLB. Previous studies did not identify an association between the severity of parkinsonism and striatal DAT uptake in DLB patients [12, 45]. These results were consistent with our results, in which no significant difference in Hoehn and Yahr score was observed between patients with DLB who had low DAT uptake and those who had normal DAT uptake. There appears to be a difference in DAT uptake in the basal ganglia between PD and DLB patients. Previous studies [12, 46] showed that patients with DLB had significantly lower DAT uptake mainly in the caudate nucleus. On the other hand, patients with PD had significantly lower DAT uptake, mainly in the putamen. Thus, these results suggested that basal ganglia pathology might differ between DLB and PD. Furthermore, in patients with DLB, multifactorial pathology may affect the nigrostriatal connection without affecting DAT binding. This was observed in a study by Liu et al., in which parkinsonism was induced by the deposition of tangles in the basal ganglia [47].

Many studies reported the coexistence of DLB and AD pathologies. Most patients with DLB also demonstrate AD pathology, including cortical amyloid plaques and neurofibrillary tangles [4, 48, 49]. On the other hand, a study by the Alzheimer’s Disease Neuroimaging Initiative reported that 45.5 % of patients with a diagnosis of AD before death also had DLB pathology [50]. O’Brien et al. reported that 63 % of patients whose diagnosis had changed from possible DLB at baseline to probable DLB at follow-up demonstrated abnormalities on DAT imaging [11]. In our study, three patients with AD had abnormal DAT uptake. Particularly for our present study, to determine whether DAT SPECT is useful for the diagnosis of DLB, we only used the clinical signs from the consortium on DLB international workshop criteria [4] for the diagnosis of DLB. Based on strict diagnostic criteria, these three patients were considered as possible DLB. Therefore, for these three patients, careful follow-up and observation of the appearance of characteristic clinical signs of DLB are necessary.

In our study, the sensitivity and specificity in differentiating DLB from AD using the H/M ratio of MIBG uptake were 72.4 and 94.4 %, respectively. Various studies have reported the diagnostic accuracy of MIBG myocardial scintigraphy. Several single-center studies [17–23] have demonstrated lower myocardial MIBG uptake in patients with DLB than in patients with the other dementias, with high sensitivity and specificity (both were approximately 90 %). The usefulness of MIBG myocardial scintigraphy in the diagnosis of DLB was recently suggested by a meta-analysis study [25]. In this study, MIBG myocardial scintigraphy demonstrated a high pooled sensitivity (98 %) and specificity (94 %) in the differential diagnosis of DLB and the other dementias. However, a recent multicenter study in Japan [24] reported a sensitivity of 68.9 % and a specificity of 87.0 % in all patients, which was consistent with our study. Moreover, this study reported that the sensitivity and specificity in university hospitals were 91.1 and 84.8 %, respectively. The variation in these results might be a result of selection bias. More typical DLB patients might have been enrolled in the single-center studies. Moreover, the fact that the mean age of patients with DLB in our study was 80.2 years, which is higher than that of the other studies, may also be a reason, because myocardial MIBG uptake is known to significantly decrease with age [51].

In our study, the patients with DLB who showed abnormalities on MIBG myocardial scintigraphy had a significantly higher frequency of RBD. Miyamoto et al. reported markedly reduced MIBG uptake in patients with idiopathic RBD, PD, and DLB [52, 53]. On the other hand, a more profound reduction in cardiac MIBG uptake was reported in patients with DLB compared with patients with PD [54]. One study [53] reported that the reduction in MIBG uptake did not significantly differ between patients with idiopathic RBD and DLB. The results of these studies using MIBG myocardial scintigraphy, including our results, suggested a strong association between RBD and DLB in Lewy body disease.

IPrevious studies [14, 15, 27–29] showed that the combined use of DAT SPECT and MIBG myocardial scintigraphy improved the diagnostic accuracy for PS and DLB. Camacho et al. showed that there was a positive association between the results of DAT SPECT and those of MIBG myocardial scintigraphy. Moreover, a close association between DAT SPECT and the presence of parkinsonism was also found [14], which is consistent with our present study. One study reported that both DAT SPECT and MIBG myocardial scintigraphy showed high diagnostic accuracy (90 %) to differentiate DLB from the other dementias [15]. Novellino et al. reported that the combined use of both DAT SPECT and MIBG myocardial scintigraphy in patients with mixed tremors and additional extrapyramidal symptoms can help differentiate patients with essential tremor from those with PD and parkinsonism [28]. Kim et al. reported that the combined use of these techniques can predict the prognosis of patients with drug-induced parkinsonism [29]. These two studies suggested that the combined use of DAT SPECT and MIBG myocardial scintigraphy is useful for the diagnosis of Lewy body disease, which is usually difficult. We categorized patients with DLB into three different groups from the results of both DAT SPECT and MIBG myocardial scintigraphy. Approximately 40 % of the patients displayed abnormalities on either DAT SPECT or MIBG myocardial scintigraphy. Moreover, parkinsonism was found at a significantly higher frequency in patients with DLB who had low DAT uptake than in those who had normal DAT uptake. On the other hand, RBD was frequently observed in patients with DLB who displayed abnormalities on MIBG myocardial scintigraphy. Patients displaying low uptake on both DAT SPECT and MIBG myocardial scintigraphy demonstrated typical clinical symptoms, suggesting an association between abnormalities on DAT SPECT or MIBG myocardial scintigraphy and clinical symptoms. Furthermore, the combined use of DAT SPECT and MIBG myocardial scintigraphy was suggested to enable the detection of patients with abnormalities on only either of the methods. However, we would like to note that for patients in whom either DAT SPECT or MIBG myocardial scintigraphy cannot be performed (e.g., DAT: patients with an infarction in the basal ganglia, patients who are unable to stop the use of medications that affect DAT uptake, etc.; MIBG: patients with heart disease or diabetes mellitus, or patients taking medications that affect MIBG uptake, etc.), the other method can be used. Our results showed that there was no significant difference between DAT SPECT and MIBG myocardial scintigraphy (*p* = 0.170) in diagnostic accuracy evaluated by ROC analysis. Moreover, when there is difficulty in diagnosing the patient after performing either of these methods, it is suggested that the other method should be performed.

This study has several critical limitations. Firstly, this study was carried out in a single memory disorder clinic; therefore, the number of patients enrolled in each treatment group was relatively small. Secondly, to determine whether DAT SPECT is useful for the diagnosis of DLB, we used the consortium on DLB international workshop criteria [4] as well as low DAT uptake in the basal ganglia. However, there appeared to be no problems in using these criteria, as all patients with DLB in this study were probable DLB patients based on the strict DLB international workshop criteria [4]. Thirdly, the DAT*MIBG index is an original index that we devised for this study. Statistical weighting was not taken into account when setting this index. Therefore, further studies are necessary to examine the validity of the DAT*MIBG index. A potential limitation of the present study is the lack of autopsy confirmation in all cases. We carefully applied rigorous standardized sets of diagnostic criteria, all of which have been shown to have a positive predictive value of greater than 80 % when judged by postmortem diagnosis [55, 56]. Further large multicenter studies, with consideration of the results of pathological examination, are required to confirm our results.

In conclusion, a combination of DAT SPECT and MIBG myocardial scintigraphy improved the sensitivity of the detection of patients with DLB. In particular, this method may provide a powerful differential diagnostic tool when it is difficult to differentiate patients with DLB from those with AD using either DAT SPECT or MIBG myocardial scintigraphy alone.
